# Gait Parameters and Peripheral Neuropathy in Patients With Diabetes: A Meta-Analysis

**DOI:** 10.3389/fendo.2022.891356

**Published:** 2022-06-01

**Authors:** Zhenchao Wang, Si Peng, Honghong Zhang, Hong Sun, Ji Hu

**Affiliations:** ^1^ Department of Endocrinology, The Second Affiliated Hospital of Soochow University, Suzhou, China; ^2^ Department of Endocrinology, Shanghai Pudong New Area People's Hospital, Shanghai, China; ^3^ Department of Traditional Chinese Medicine, The Second Affiliated Hospital of Soochow University, Suzhou, China

**Keywords:** diabetes mellitus, DPN, gait parameters, gait abnormalities, meta-analysis

## Abstract

**Objective:**

To investigate the relationship between diabetic peripheral neuropathy (DPN) and gait abnormality in diabetic patients.

**Methods:**

Related research concerning the gait of diabetic patients with and without DPN was collected and analyzed by searching PubMed, Embase, and Web of Science. Statistical analysis was performed by using RevMan 5.3 software.

**Results:**

Twenty-one studies were included in this meta-analysis, consisting of 499 diabetic neuropathy patients and 467 diabetes controls without neuropathy. Meta-analysis results showed lower gait velocity, shorter stride length, longer stride time, longer stance time, and higher maximum knee extension moment in the DPN group, compared with their counterparts.

**Conclusion:**

Among diabetic patients, those complicated with DPN possess lower gait velocity, shorter stride length, longer stride time, longer stance time, and higher maximum knee extension moment.

## Introduction

As a common endocrine disease featured by impaired glucose metabolism, diabetes is afflicting approximately 10% of the adult population worldwide (1). Furthermore, the long-term hyperglycemic condition in patients can damage many target organs like the nerves, retina, and kidney, inducing various complications such as diabetic nephropathy, diabetic retinopathy, and diabetic nephropathy, respectively. Diabetic nephropathy can be further divided into diabetic central neuropathy and diabetic peripheral neuropathy (DPN), and the latter is more common in clinical practice. As one of the most common complications of diabetes, DPN affects about half of diabetes worldwide (2). The Toronto Consensus Panel on Diabetic Neuropathy defined DPN as a “symmetrical, length-dependent sensorimotor polyneuropathy attributable to metabolic and micro-vessel alterations as a result of chronic hyperglycemic exposure and cardiovascular risk covariates” (3). It is usually characterized as sensory or motor neuron abnormalities, which could lead to morbidity and mortality in patients and inevitably exacerbate their suffering, decreasing their quality of life. In addition, DPN patients often have gait abnormalities due to protective sensory impairment, reduced muscle power, and other reasons, resulting in falls and diabetic foot. However, many individuals are not diagnosed with DPN timely and thus usually fail to receive prompt intervention (4).

Recently, the association between DPN and its influence on human gait has been more and more highlighted. It has been reported that the development of DPN can lead to the dysfunction of muscle (5). Walking, as a natural daily activity that relies heavily on the cooperation of the nervous system and musculoskeletal system, can be easily affected by the pathological process in DPN patients (6). The alteration of the neuromuscular system can be a significant factor in gait variability. Because peripheral neuropathy is also found in pre-diabetes (7), gait analysis can be helpful for timely intervention in the diabetic population.

Therefore, this meta-analysis was conducted to review the published studies focusing on the characteristics of gait parameters in diabetics with and without peripheral neuropathy, hoping that more attention could be paid to the gait abnormality in DPN patients.

## Methods

### Search Strategy

A systematic search of PubMed, Embase, and Web of Science was conducted from inception to September 2020. The following keywords and MeSH headings were used:

1.exp diabetic neuropathy/
DPN
diabetic peripheral neuropath*
1 or 2 or 3
exp gait/
exp kinematics/
gait parameter*
gait disorders
gait characteristic*
gait analysis
or/5-10
4 and 11
limit 12 to humans

### Study Selection Process and Criteria

**Figure 1:**
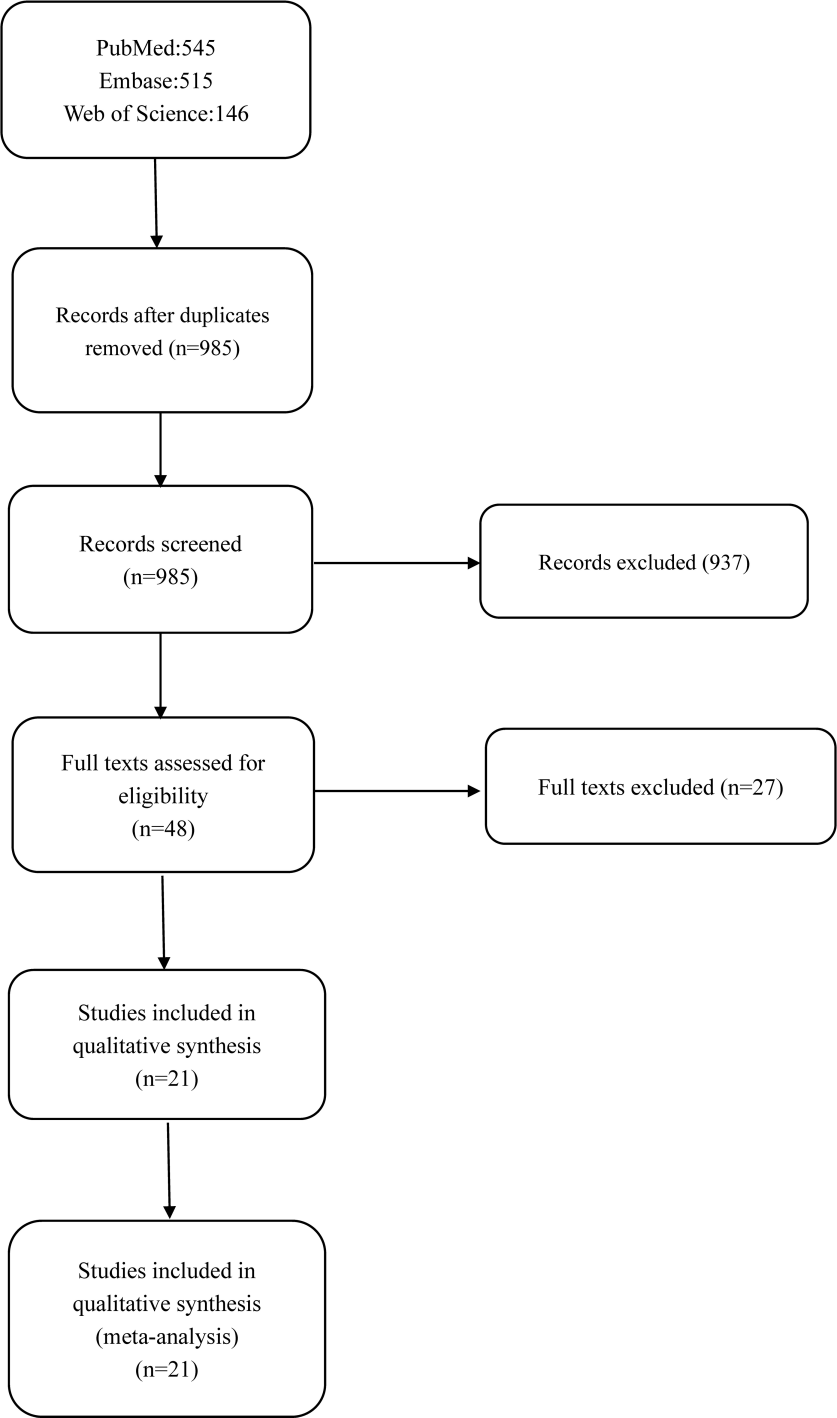
Flow diagram of studies selection.

A total of 1,205 studies were obtained using all the search engines mentioned above, including PubMed (n = 545), Embase (n = 515), and Web of Science (n = 146). The duplicates were removed, and finally, 21 studies were selected (8–28) (based on the inclusion and exclusion criteria below). The selection process and records have been diagrammatically shown in **Figure 1**.

**Table:** 

Inclusion criteria	Exclusion criteria
1. Studies comparing diabetic patients with and without neuropathy (DPN and DC groups)	1. Studies included current or past diabetes foot ulcer participants as a part of their DPN or DC groups
2. Observational studies in the English language	2. Studies performed on a treadmill or the walking speed is imposed
3. Studies reported outcomes for at least one of the following: gait velocity, stride length, stride time, stance time, range of motion (RoM) in the sagittal plane of the knee joint, and the maximum knee extension moment.	3. Studies in which the outcomes cannot be compared or the key data are unmentioned
4. The classification of participants is based on the objective diagnostic criteria	4. Studies performed on uneven or obstacle roads

### Data Extraction

Two authors extracted the data independently according to predefined criteria. A difference of opinion between the two authors was resolved through discussion. The third author made the final decision if there is any disagreement. The data including the first author, year of publication, age of participants, observed outcomes, and quality assessment were extracted. Our outcome measures included gait velocity, stride length, stride time, stance time, stance time proportion, RoM in the sagittal plane of the knee joint, and the maximum knee extension moment. Data of the subgroup were merged when necessary.

### Quality Assessment

Two authors independently assessed the quality of the included articles according to the article quality assessment tool. Twenty cross-sectional studies were evaluated by the Agency for Healthcare Research and Quality scale (AHRQ scale), and one self-control study was assessed by Methodological Index for Non-randomized Studies (MINORS). Any disagreement was resolved by discussion. The AHRQ scale consists of 11 items that assess the quality of study by the answer “yes = 1” or “no = 0” or “uncertain = 0.” Quality was assigned as low with a total score ≤3, high quality with a total score ≥8, and medium quality in other cases. The MINORS contains 12 items. Each item was scored from 0 to 2, 0 indicating that it was not reported in the article evaluated, 1 indicating that it was reported but inadequately, and 2 indicating that it was reported adequately. If a study’s total MINORS score is under 13, it will be regarded as low quality.

### Statistical Analysis

All data were analyzed by RevMan 5.3 software. Since all the data included in this meta-analysis are continuous, weighted mean difference (WMD) and 95% CI are calculated if the outcome shares the same units of measurement, or the standardized mean difference (SMD) was used instead. Statistical heterogeneity was evaluated by the *I* ^2^ test. The fixed-effect model was used if *I* ^2^ < 50%, and the random-effects model was used if *I* ^2^ ≥ 50%. Publication bias was detected by funnel plot.

## Results

### Search Yield and Study Characteristics

**Figure 1** outlines the process and results of each step of the literature search. A total of 1,205 articles were initially identified. However, 1,185 articles were excluded for a variety of reasons, such as lack of DC groups, imposed walking speed, inappropriate methods used in data capture, irrelevant data, or the desired data cannot be calculated. As a result, 21 articles remained eligible for inclusion, comprising 20 cross-sectional studies and one self-control study. Among them, gait velocity was reported in all 21 studies, stride length in 11 studies, stride time in 7 studies, stance time in 6 studies, the proportion of stance time in gait cycle in 3 studies, RoM in the sagittal plane of the knee joint in 5 studies, and the maximum knee extension moment was reported in 3 studies. The general characteristics of the 21 studies and the quality scores are summarized in **Table 1**.

**Table 1:** Overview and quality assessment of considered studies.

Study	Method	DPN group		DC group		Examined variables	AHRQ(MINORS) score
		n	Age (mean ± SD)	n	Age (mean ± SD)		
Allet, 2009 (8)	Portable gait analysis system	15	61.29 (6.52)	15	55.83 (8.20)	①②③⑤⑥	6
Brown, 2014 (9)	3D/2D gait analysis system	20	57 (9)	33	58 (12)	①④⑦	5
Brown, 2015 (10)	3D/2D gait analysis system	22	57 (9)	39	56 (13)	①	6
Merriwether EN, 2016 (11)	3D/2D gait analysis system	11	63 (11)	12	58 (9)	①	6
Guiotto, 2013 (12)	3D/2D gait analysis system	20	60.30 (9.60)	20	62.90 (5.63)	①②③④	5
Hastings, 2014 (13)	3D/2D gait analysis system	12	59 (13)	12	58 (8)	①	6
Katoulis, 1997 (14)	3D/2D gait analysis system	20	52.9 (8.8)	20	47.6 (10.7)	①	7
Kelly, 2013 (15)	Portable gait analysis system	16	73 (8)	18	62 (7)	①②③	7
Melai-2013 (16)	Plantar pressure pad	94	66.9 (7.5)	39	62.4 (6.6)	①②③④⑦	6
Morrison S, 2014 (17)	Plantar pressure pad	16	61.1 (1.9)	21	58.7 (1.8)	①②⑤	18
Paul, 2009 (18)	Plantar pressure pad	15	69 (3.0)	15	70 (2.9)	①	7
Petrovic, 2018 (19)	3D/2D gait analysis system	13	61 (7)	20	57 (8)	①⑥	5
Raspovic, 2013 (20)	3D/2D gait analysis system	10	64 (6.4)	10	59 (10.5)	①②⑥	7
Roman de Mettelinge T, 2013 (21)	Portable gait analysis system	28	74.8 (7.5)	28	74.1 (8.2)	①②	5
Savelberg, 2010 (22)	3D/2D gait analysis system	8	68.9 (6.3)	10	60.5 (6.9)	①②③④	6
Sawacha, 2010 (23)	3D/2D gait analysis system	26	63.2 (6.0)	20	63.8 (5.4)	①②③④⑥	5
Sawacha, 2012 (24)	3D/2D gait analysis system	20	61.2 (7.7)	20	56.53 (13.29)	①②③④⑤	6
Shaw, 1998 (25)	3D/2D gait analysis system	51	54.5 (10.9)	60	52.3 (9.9)	①	5
Suda, 2019 (26)	3D/2D gait analysis system	43	57.7 (5.6)	26	59.1 (5.1)	①②	5
Williams, 2007 (27)	3D/2D gait analysis system	10	49.5 (3.7)	12	51.1 (10.9)	①	6
Yavuzer, 2006 (28)	3D/2D gait analysis system	20	61.7 (8.5)	26	58.2 (9.5)	①⑥⑦	6

①, gait velocity (m/s); ②, stride length (m); ③, stride time (s); ④, stance time (s); ⑤, stance time (%); ⑥, RoM in sagittal plane of knee joint; ⑦, maximum knee extension moment.

DPN, diabetic peripheral neuropathy; DC, diabetes control; AHRQ, Agency for Healthcare Research and Quality; MINORS, Methodological Index for Non-randomized Studies.

### Meta-Analysis of Outcomes

#### Gait Velocity (m/s)

All of the 21 studies mentioned above observed the gait velocity of both the DPN and DC groups. The meta-analysis performed on gait velocity showed that there was a significantly lower gait velocity in DPN patients compared to their counterparts (SMD: −0.28, 95% CI −0.41 to −0.14; *p* < 0.0001; *I* ^2^ = 37%).

#### Stride Length (m)

Of the included studies, 11 observed the stride length of the DPN and DC groups. The meta-analysis showed that the stride length in DPN patients is shorter than that of their counterparts (SMD: −0.19, 95% CI −0.36 to −0.01; *p* = 0.04; *I* ^2^ = 39%).

#### Stride Time (s)

Seven studies observed stride time (s). The combined data indicated that stride time in the DPN group was significantly longer than that of the DC group (WMD = 0.04, 95% CI 0.01 to 0.06; *p* = 0.006; *I* ^2^ = 0%).

#### Stance Time (s)

Six studies reported their observations on the stance time of the participants. The meta-analysis showed that the DPN group tends to have a longer stance phase in the gait cycle (WMD = 0.03, 95% CI 0.01 to 0.05; *p* = 0.002; *I* ^2^ = 12%).

#### Stance Time Proportion (%)

Three studies observed stance time proportion (%) in their research. The meta-analysis showed that DPN patients had longer percentage duration in the stance phase of gait (WMD = 1.02, 95% CI 0.16 to 1.89; *p* = 0.02; *I* ^2^ = 0%).

#### Range of Motion of Knee Joint in Sagittal Plane (°)

Five studies recorded the range of motion in the sagittal plane of the knee joint as a kinematic parameter in their research. The meta-analysis result showed that there was no statistically significant difference between the two groups in this parameter (WMD = −0.44, 95% CI −4.31 to 3.43; *p* = 0.82; *I* ^2^ = 53%).

#### Maximum Knee Extension Moment (N·m/kg)

Three studies recorded the maximum knee extension moment as a kinetic parameter in their research. The meta-analysis suggested that this parameter was higher in the DPN group compared to the DC group (WMD = 0.0675, 95% CI 0.0038 to 0.1312; *p* = 0.04; *I* ^2^ = 0%).

### Publication Bias

**Figure 2:**
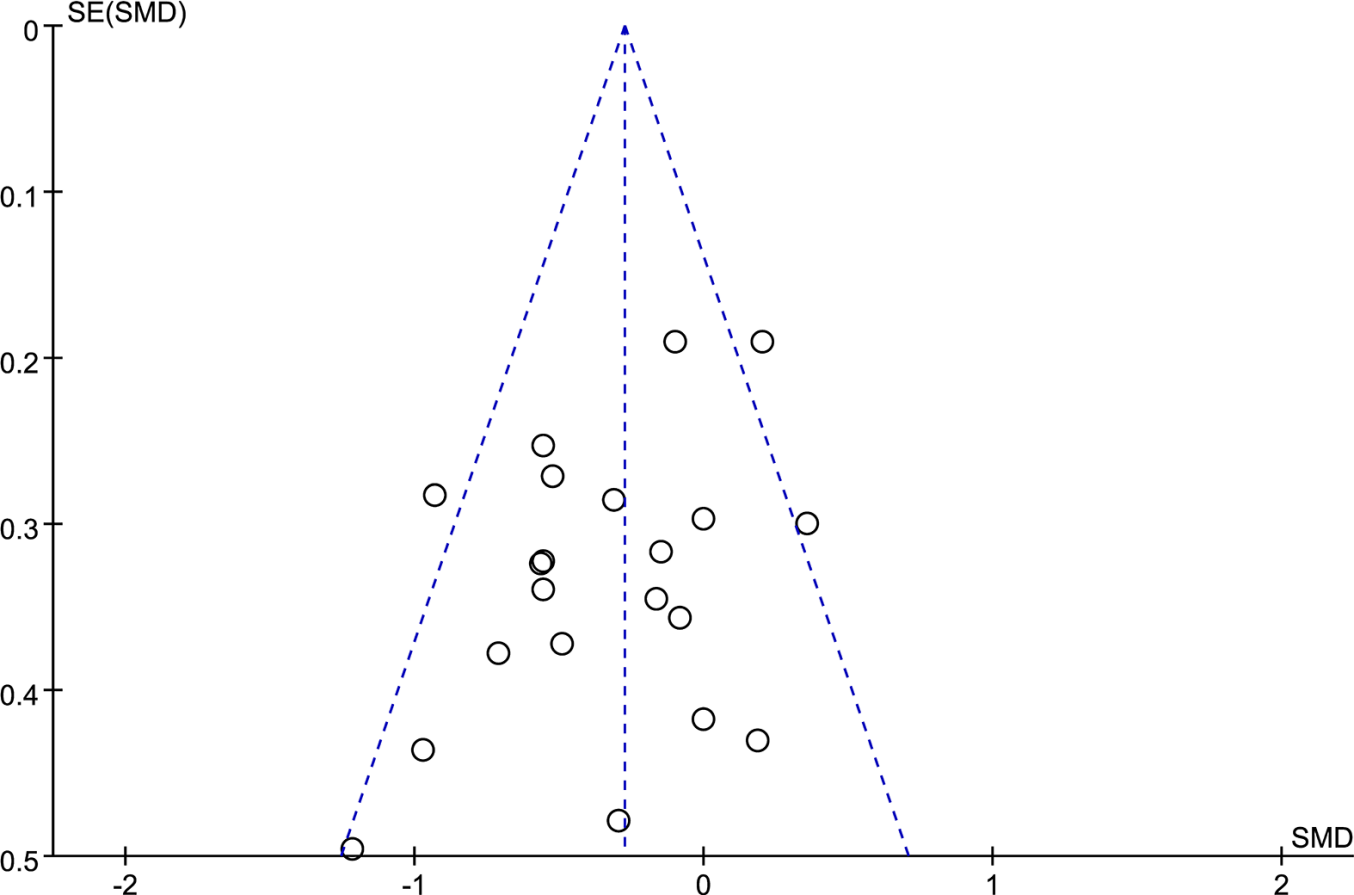
Funnel plot of gait velocity.

A funnel plot (**Figure 2**) was used in this meta-analysis to detect potential publication bias in the outcome of gait velocity. The symmetric distribution in the funnel plot demonstrated no potential publication bias in the studies.

## Discussion

**Figure 3:**
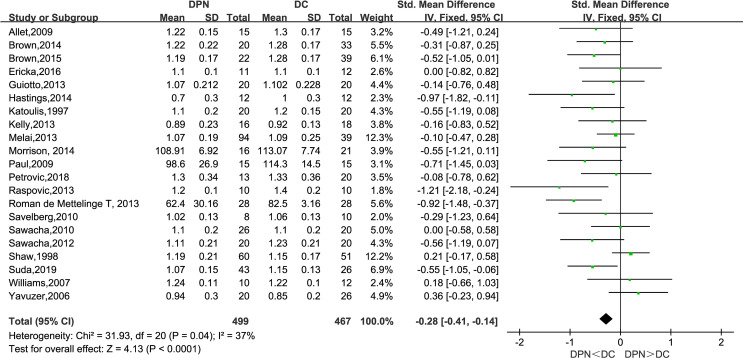
Forest plot of gait velocity(m/s).

**Figure 4:**
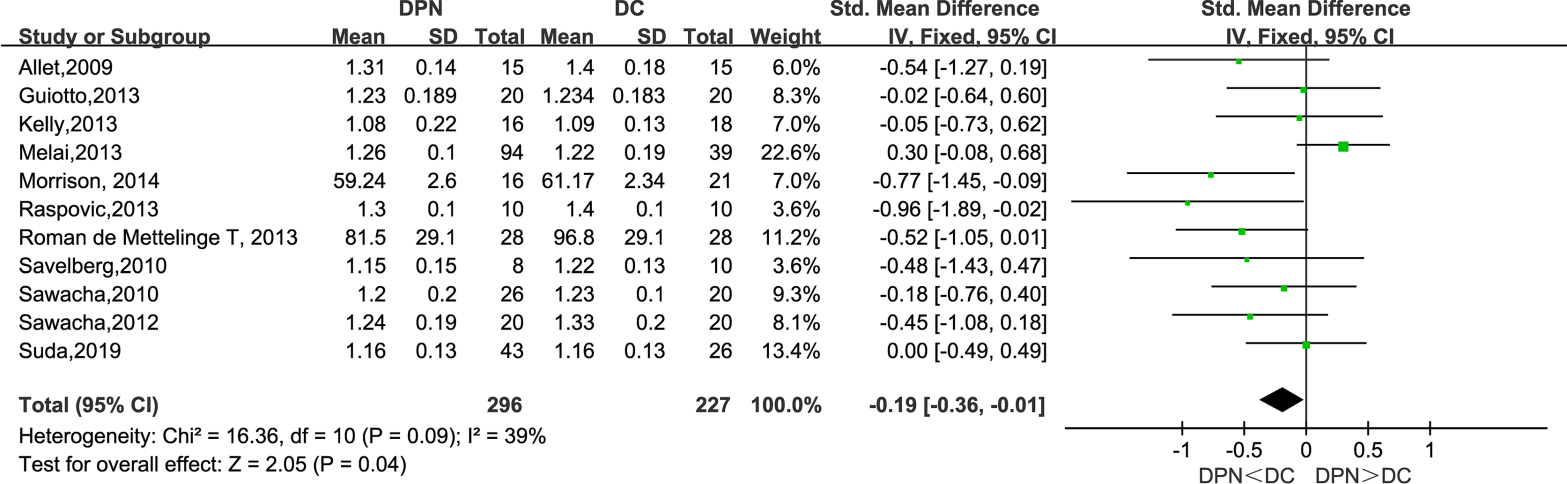
Forest plot of stride length(m).

**Figure 5:**
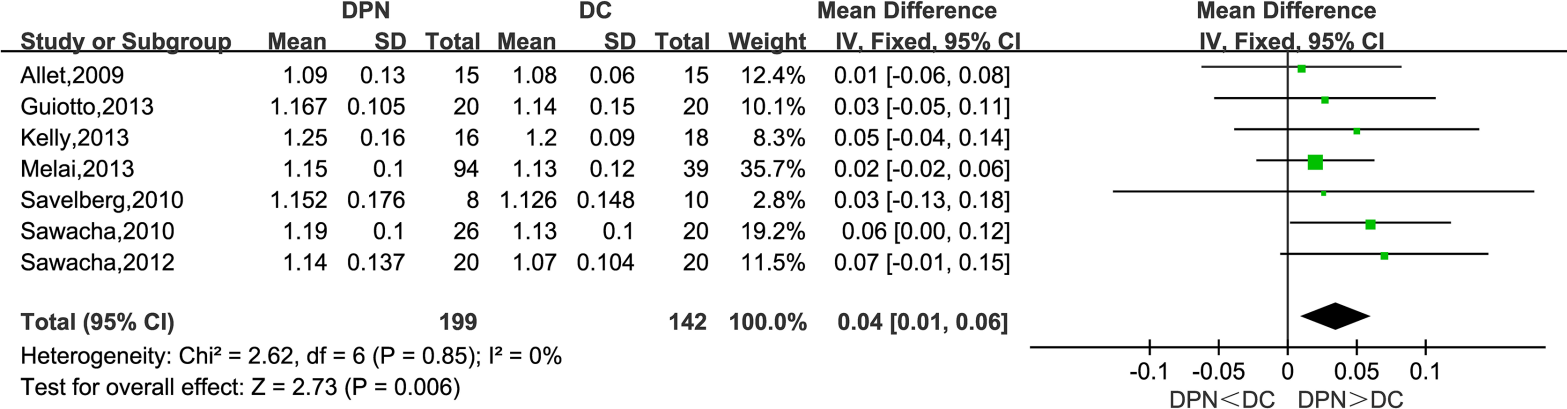
Forest plot of stride time(s).

**Figure 6:**
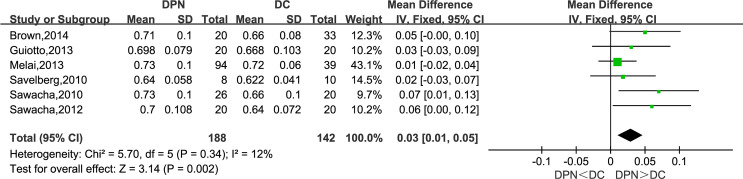
Forest plot of stance time(s).

**Figure 7:**
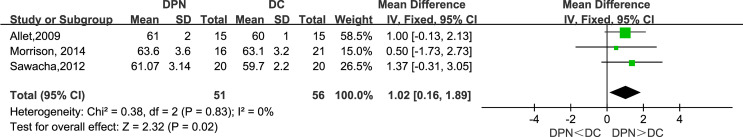
Forest plot of stance time(%).

**Figure 8:**
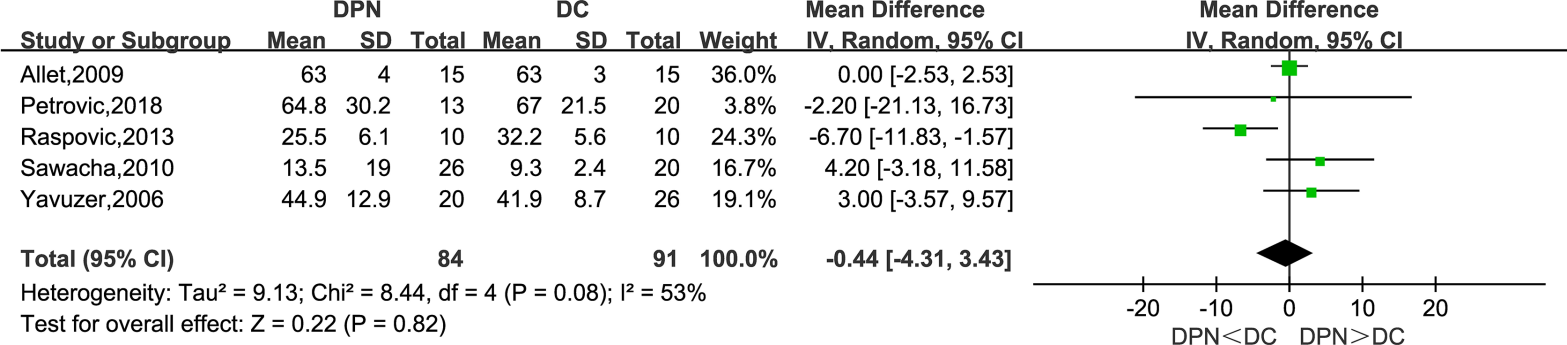
Forest plot of RoM of knee in sagittal plane(°).

**Figure 9:**
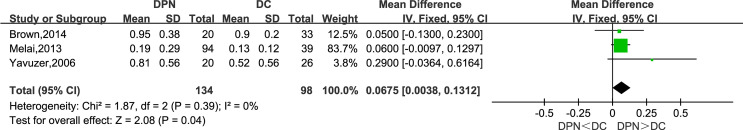
Forest plot of maximum knee extension moment(N·m/kg).

Consistent with previous studies, this meta-analysis unveiled a significant difference between diabetic patients with and without neuropathy in spatiotemporal gait parameters (29). As is shown in the forest plot above (**Figures 3**–**9**), diabetic patients complicated with neuropathy walked at slower speed, shorter stride length, longer stride time, longer stance time, and higher maximum knee extension moment.

DPN patients may experience symptoms such as paresthesia and numbness (30). Notably, almost 50% of people with DPN suffer from peripheral neuropathic pain (31). These symptoms can finally lead to gait abnormality.

Many researchers reckon that the early or less severe stages of DPN typically result in sensory impairments. The development of the disease can eventually lead to major dysfunction of the neuromuscular system. These impairments may include muscle atrophy and weakness, slowing of muscle contraction, and loss of power and endurance. As a result, the loss of dorsiflexion mobility is seen as a key factor resulting in lower speed in DPN patients (32). Finally, the continuously pathologic processes of DPN can contribute to altered gait and increased fall risk (33).

Except for the explanations mentioned above, the central nervous system (CNS) also contributes to the gait abnormality in DPN patients. Epidemiologic studies have reported that diabetes is an independent risk factor for cognitive impairment (34, 35). Previous research indicated that the CNS can be affected by both the metabolic and vascular consequences of diabetes, causing cognitive dysfunction and neurologic impairment (36). Therefore, the information processing speed of CNS declines (37), which may cause conservative gait patterns in DPN individuals. Additionally, impairment of protective sensation toward pain and high pressure on the forefoot makes it easier for neuropathies to develop ulceration. That, in turn, causes abnormal gait (38).

It is also worth mentioning that the DPN group shows a higher maximum knee extension moment, but a clear explanation is yet to come. The following explanation may be helpful. At the early stage of the stance phase in the gait cycle, with the heel as the fulcrum, the tibia moves forward faster than the femur. At this time, an extension torque is needed to maintain the stability of knee joints, and the quadriceps femoris contracts to provide this torque. So the increase of peak knee extension torque indicates that the patients with DPN have to pay a higher price to keep their balance. In addition, the RoM in the sagittal plane of the knee joint between the two groups shows no significant difference. The reasons may be that the knee joint is controlled by many large muscles possessing enough compensative capacity, and DPN mainly affects distal limbs. So it may not be a suitable indicator of the gait abnormality caused by DPN.

We need to acknowledge some limitations in this study. First, most of the studies included in this meta-analysis do not view diabetic retinopathy as part of the exclusion criteria. However, as another common diabetic complication, diabetic retinopathy could also contribute to gait abnormality (39). Second, the outcomes we selected are limited. Despite these limitations, our meta-analysis has many advantages. First, many of our combined results show a low heterogeneity. Second, quite a number of studies are included in this meta-analysis.

## Conclusion

In summary, this meta-analysis unveils that there exists a significant difference between diabetic patients complicated with DPN and the diabetic controls in gait parameters, including gait velocity, stride length, stride time, stance time, and the maximum knee extension moment. These deviations may be related to the development of peripheral polyneuropathy, low limb muscle dysfunction, and cognitive impairment and may be an explanation for fall events among DPN patients. More research is needed to confirm and update the findings of this analysis.

## Data Availability Statement

The original contributions presented in the study are included in the article/supplementary material, further inquiries can be directed to the corresponding authors.

## Funding

This work was supported by the National Natural Science Foundation of China (No. 82170836 and No. 82071234).

## Conflict of Interest

The authors declare that the research was conducted in the absence of any commercial or financial relationships that could be construed as a potential conflict of interest.

## Publisher’s Note

All claims expressed in this article are solely those of the authors and do not necessarily represent those of their affiliated organizations, or those of the publisher, the editors and the reviewers. Any product that may be evaluated in this article, or claim that may be made by its manufacturer, is not guaranteed or endorsed by the publisher.

## References

[1] Sun H Saeedi P Karuranga S Pinkepank M Ogurtsova K Duncan BB . IDF Diabetes Atlas: Global, Regional and Country-Level Diabetes Prevalence Estimates for 2021 and Projections for 2045. Diabetes Res Clin Pract (2022) 183:109119. doi: 10.1016/j.diabres.2021.109119 34879977 PMC11057359

[2] Iqbal Z Azmi S Yadav R Ferdousi M Kumar M Cuthbertson DJ . Diabetic Peripheral Neuropathy: Epidemiology, Diagnosis, and Pharmacotherapy. Clin Ther (2018) 40(6):828–49. doi: 10.1016/j.clinthera.2018.04.001 29709457

[3] Tesfaye S Boulton AJ Dyck PJ Freeman R Horowitz M Kempler P . Diabetic Neuropathies: Update on Definitions, Diagnostic Criteria, Estimation of Severity, and Treatments. Diabetes Care (2010) 33:2285–93. doi: 10.2337/dc10-1303 PMC2945176 20876709

[4] Tesfaye S . Recent Advances in the Management of Diabetic Distal Symmetrical Polyneuropathy. J Diabetes Investig (2011) 2(1):33–42. doi: 10.1111/j.2040-1124.2010.00083.x PMC4008012 24843458

[5] Moore CW Allen MD Kimpinski K Doherty TJ Rice CL . Reduced Skeletal Muscle Quantity and Quality in Patients With Diabetic Polyneuropathy Assessed by Magnetic Resonance Imaging. Muscle Nerve (2016) 53(5):726–32. doi: 10.1002/mus.24779 26202052

[6] Bonnet C Carello C Turvey MT . Diabetes and Postural Stability: Review and Hypotheses. J Mot Behav (2009) 41(2):172–92. doi: 10.3200/jmbr.41.2.172-192 19201687

[7] Stino AM Smith AG . Peripheral Neuropathy in Prediabetes and the Metabolic Syndrome. J Diabetes Invest (2017) 8(5):646–55. doi: 10.1111/jdi.12650 PMC5583955 28267267

[8] Allet L Armand S de Bie RA Pataky Z Aminian K Herrmann FR . Gait Alterations of Diabetic Patients While Walking on Different Surfaces. Gait Posture (2009) 29(3):488–93. doi: 10.1016/j.gaitpost.2008.11.012 19138520

[9] Brown SJ Handsaker JC Bowling FL Maganaris CN Boulton AJ Reeves ND . Do Patients With Diabetic Neuropathy Use a Higher Proportion of Their Maximum Strength When Walking? J Biomech (2014) 47(15):3639–44. doi: 10.1016/j.jbiomech.2014.10.005 25458154

[10] Brown SJ Handsaker JC Bowling FL Boulton AJ Reeves ND . Diabetic Peripheral Neuropathy Compromises Balance During Daily Activities. Diabetes Care (2015) 38(6):1116–22. doi: 10.2337/dc14-1982 25765355

[11] Merriwether EN Hastings MK Mueller MJ Bohnert KL Strube MJ Snozek DR . Static and Dynamic Predictors of Foot Progression Angle in Individuals With and Without Diabetes Mellitus and Peripheral Neuropathy. Ann Gerontol Geriatr Res (2016) 3(2):1038. doi: 10.5348/D05-2016-7-OA-3 27882360 PMC5117663

[12] Guiotto A Sawacha Z Guarneri G Cristoferi G Avogaro A Cobelli C . The Role of Foot Morphology on Foot Function in Diabetic Subjects With or Without Neuropathy. Gait Posture (2013) 37(4):603–10. doi: 10.1016/j.gaitpost.2012.09.024 23159679

[13] Hastings MK Woodburn J Mueller MJ Strube MJ Johnson JE Beckert KS . Radiographic-Directed Local Coordinate Systems Critical in Kinematic Analysis of Walking in Diabetes-Related Medial Column Foot Deformity. Gait Posture (2014) 40(1):128–33. doi: 10.1016/j.gaitpost.2014.03.010 PMC4038905 24703359

[14] Katoulis EC Ebdon-Parry M Lanshammar H Vileikyte L Kulkarni J Boulton AJ . Gait Abnormalities in Diabetic Neuropathy. Diabetes Care (1997) 20(12):1904–7. doi: 10.2337/diacare.20.12.1904 9405916

[15] Kelly C Fleischer A Yalla S Grewal GS Albright R Berns D . Fear of Falling is Prevalent in Older Adults With Diabetes Mellitus But is Unrelated to Level of Neuropathy. J Am Podiatr Med Assoc (2013) 103(6):480–8. doi: 10.7547/1030480 PMC4732269 24297984

[16] Melai T Schaper NC Ijzerman TH de Lange TL Willems PJ Meijer K . Increased Forefoot Loading is Associated With an Increased Plantar Flexion Moment. Hum Mov Sci (2013) 32(4):785–93. doi: 10.1016/j.humov.2013.05.001 23958476

[17] Morrison S Colberg SR Parson HK Vinik AI . Exercise Improves Gait, Reaction Time and Postural Stability in Older Adults With Type 2 Diabetes and Neuropathy. J Diabetes Complications (2014) 28(5):715–22. doi: 10.1016/j.jdiacomp.2014.04.007 24929798

[18] Paul L Ellis BM Leese GP McFadyen AK McMurray B . The Effect of a Cognitive or Motor Task on Gait Parameters of Diabetic Patients, With and Without Neuropathy. Diabetic Med (2009) 26(3):234–9. doi: 10.1111/j.1464-5491.2008.02655.x 19317817

[19] Petrovic M Maganaris CN Deschamps K Verschueren SM Bowling FL Boulton AJM . Altered Achilles Tendon Function During Walking in People With Diabetic Neuropathy: Implications for Metabolic Energy Saving. J Appl Physiol (2018) 124(5):1333–40. doi: 10.1152/japplphysiol.00290.2017 29420151

[20] Raspovic A . Gait Characteristics of People With Diabetes-Related Peripheral Neuropathy, With and Without a History of Ulceration. Gait Posture (2013) 38(4):723–8. doi: 10.1016/j.gaitpost.2013.03.009 23583607

[21] Roman de Mettelinge T Delbaere K Calders P Gysel T Van DenNoortgate N Cambier D . The Impact of Peripheral Neuropathy and Cognitive Decrements on Gait in Older Adults With Type 2 Diabetes Mellitus. Arch Phys Med Rehabil (2013) 94(6):1074–9. doi: 10.1016/j.apmr.2013.01.018 23385112

[22] Savelberg HHCM Ilgin D Angin S Willems PJ Schaper NC Meijer K . Prolonged Activity of Knee Extensors and Dorsal Flexors is Associated With Adaptations in Gait in Diabetes and Diabetic Polyneuropathy. Clin Biomech (2010) 25(5):468–75. doi: 10.1016/j.clinbiomech.2010.02.005 20207058

[23] Sawacha Z Guarneri G Avogaro A Cobelli C . A New Classification of Diabetic Gait Pattern Based on Cluster Analysis of Biomechanical Data. J Diabetes Sci Technol (2010) 4(5):1127–38. doi: 10.1177/193229681000400511 PMC2956820 20920432

[24] Sawacha Z Spolaor F Guarneri G Contessa P Carraro E Venturin A . Abnormal Muscle Activation During Gait in Diabetes Patients With and Without Neuropathy. Gait Posture (2012) 35(1):101–5. doi: 10.1016/j.gaitpost.2011.08.016 22098824

[25] Shaw JE van Schie CH Carrington AL Abbott CA Boulton AJ . An Analysis of Dynamic Forces Transmitted Through the Foot in Diabetic Neuropathy. Diabetes Care (1998) 21(11):1955–9. doi: 10.2337/diacare.21.11.1955 9802750

[26] Suda EY Matias AB Bus SA Sacco ICN . Impact of Diabetic Neuropathy Severity on Foot Clearance Complexity and Variability During Walking. Gait Posture (2019) 74:194–9. doi: 10.1016/j.gaitpost.2019.09.014 31550557

[27] Williams DS 3rd Brunt D Tanenberg RJ . Diabetic Neuropathy is Related to Joint Stiffness During Late Stance Phase. J Appl Biomech (2007) 23(4):251–60. doi: 10.1123/jab.23.4.251 18089923

[28] Yavuzer G Yetkin I Toruner FB Koca N Bolukbasi N . Gait Deviations of Patients With Diabetes Mellitus: Looking Beyond Peripheral Neuropathy. Eura Medicophys (2006) 42(2):127–33. PMID(16767059) 16767059

[29] Hazari A Maiya AG Shivashankara KN Agouris I Monteiro A Jadhav R . Kinetics and Kinematics of Diabetic Foot in Type 2 Diabetes Mellitus With and Without Peripheral Neuropathy: A Systematic Review and Meta-Analysis. SpringerPlus (2016) 5(1):1819. doi: 10.1186/s40064-016-3405-9 27812455 PMC5071310

[30] Galer BS Gianas A Jensen MP . Painful Diabetic Polyneuropathy: Epidemiology, Pain Description, and Quality of Life. Diabetes Res Clin Pract (2000) 47:123–8. doi: 10.1016/S0168-8227(99)00112-6 10670912

[31] Alleman CJ Westerhout KY Hensen M Chambers C Stoker M Long S . Humanistic and Economic Burden of Painful Diabetic Peripheral Neuropathy in Europe: A Review of the Literature. Diabetes Res Clin Pract (2015) 109:215–25. doi: 10.1016/j.diabres.2015.04.031 26008721

[32] Martinelli AR Mantovani AM Nozabieli AJ Ferreira DM Barela JA Camargo MR . Muscle Strength and Ankle Mobility for the Gait Parameters in Diabetic Neuropathies. Foot (2013) 23(1):17–21. doi: 10.1016/j.foot.2012.11.001 23274122

[33] Allen MD Doherty TJ Rice CL Kimpinski K . Physiology in Medicine: Neuromuscular Consequences of Diabetic Neuropathy. J Appl Physiol (2016) 121(1):1–6. doi: 10.1152/japplphysiol.00733.2015 26989220 PMC4967246

[34] Allen KV Frier BM Strachan MW . The Relationship Between Type 2 Diabetes and Cognitive Dysfunction: Longitudinal Studies and Their Methodological Limitations. Eur J Pharmacol (2004) 490(1-3):169–75. doi: 10.1016/j.ejphar.2004.02.054 15094083

[35] Stewart R Liolitsa D . Type 2 Diabetes Mellitus, Cognitive Impairment and Dementia. Diabetic Med (1999) 16(2):93–112. doi: 10.1046/j.1464-5491.1999.00027.x 10229302

[36] Selvarajah D Tesfaye S . Central Nervous System Involvement in Diabetes Mellitus. Curr Diabetes Rep (2006) 6(6):431–8. doi: 10.1007/s11892-006-0075-y 17118225

[37] Sommerfield AJ Deary IJ Frier BM . Acute Hyperglycemia Alters Mood State and Impairs Cognitive Performance in People With Type 2 Diabetes. Diabetes Care (2004) 27(10):2335–40. doi: 10.2337/diacare.27.10.2335 15451897

[38] Karmakar S Rashidian H Chan C Liu C Toth C . Investigating the Role of Neuropathic Pain Relief in Decreasing Gait Variability in Diabetes Mellitus Patients With Neuropathic Pain: A Randomized, Double-Blind Crossover Trial. J Neuroeng Rehabil (2014) 11(1):125. doi: 10.1186/1743-0003-11-125 25139539 PMC4150964

[39] Khalaf K Al-Angari HM Khandoker AH Lee S Almahmeed W Al Safar HS . Gait Alterations in the UAE Population With and Without Diabetic Complications Using Both Traditional and Entropy Measures. Gait Posture (2017) 58:72–7. doi: 10.1016/j.gaitpost.2017.07.109 28756345

